# Flow Electrolysis on Anodized Carbon Fibers for Pu Separation and Analysis

**DOI:** 10.1021/acs.analchem.5c00496

**Published:** 2025-05-13

**Authors:** Paul Dutheil, Fabian Köhler, Martin Heule, Noemi Cerboni, Patrick Steinegger

**Affiliations:** † Department of Chemistry and Applied Biosciences, ETH Zürich, Leopold-Ruzicka-Weg 4, Zürich CH-8093, Switzerland; ‡ Department of Radiation Safety and Security, Paul Scherrer Institute, Forschungsstrasse 111, Villigen PSI CH-5232, Switzerland; § PSI Center for Nuclear Engineering and Sciences, Paul Scherrer Institute, Forschungsstrasse 111, Villigen PSI CH-5232, Switzerland

## Abstract

The analysis of Pu isotopes with radiometric or mass spectrometry techniques requires prior chemical separation to overcome interferences from other actinides and to remove matrix components. These separations are usually carried out using extraction or ion exchange chromatography. In this work, flow electrolytic separation on anodized carbon fibers is explored as a new alternative approach for the separation and analysis of Pu isotopes by means of radiometric methods. A high-surface-area carbon fiber felt electrode was anodized and used for flow electrolytic accumulation and release of Pu. Characterization of the anodized carbon fiber felt was carried out using X-ray photoelectron and infrared spectroscopy. The conditions needed for the retention of Pu during electrolysis were investigated, optimized, and used to develop a method for the measurement of ^238^Pu or ^239,240^Pu by α-spectrometry. This method was evaluated with digested solid samples (i.e., wipe test, ceramics, and sludge) and compared with traditional chromatographic separation approaches. It was found that oxygen-containing functional groups are introduced on the carbon fiber surface upon its anodization. This allows the accumulation of Pu­(IV), which is produced by adjusting the electrode potential and can be released by electroreduction to Pu­(III), whereas other actinides (e.g., U, Am, and Cm) as well as matrix components are not retained. This provides a fast, single-step separation of Pu, free of impurities from reagents or resins, which may be detrimental to the preparation of α-sources. The successful measurement of Pu isotopes confirmed the reliability and good tolerance of this approach for highly complex matrices.

## Graphical abstract

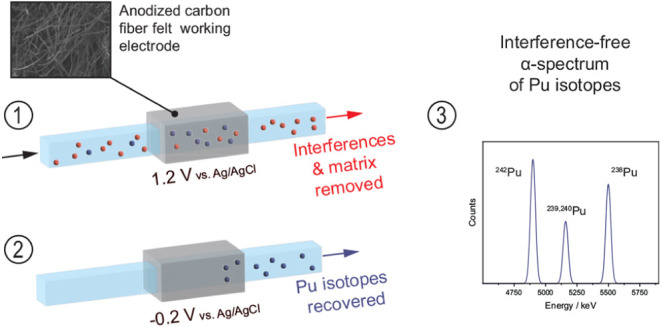

## Introduction

Plutonium is ubiquitous in the nuclear industry due to its high technological importance for nuclear energy production and its uses in nuclear weapons or for nuclear propulsion. However, the toxicity of Pu, even in low amounts, together with the long half-lives of its isotopes and its persistence in the environment, makes it a concerning contaminant.^1^ As a result, Pu isotopes must be measured in order to ensure the safe operation and decommissioning of nuclear facilities, not only in the context of radioactive waste management and storage but also in the framework of environmental monitoring, and emergency response.^2^ Furthermore, the potential nonpeaceful applications of Pu render its analysis as highly important for nuclear safeguards^3^ and nuclear forensics.^4,5^ Therefore, the radioactivity concentration of Pu isotopes (especially ^238^Pu, ^239^Pu, ^240^Pu, and ^241^Pu) and their ratios need to be reliably measured in a wide variety of samples in support of nuclear research and industry, as well as regulatory bodies and authorities.

Depending on their half-lives and decay types, Pu isotopes are either measured by radiometric methods such as α-spectrometry and liquid scintillation counting (LSC)^2,6^ or by inorganic mass spectrometry techniques, e.g., inductively coupled plasma mass spectrometry (ICP-MS), thermal ionization mass spectrometry (TIMS), and secondary ion mass spectrometry (SIMS).^5,7,8^ Pu isotopes are often found in the presence of other actinides such as U, Am, Cm, or Th, as well as various matrix elements. Actinide isotopes aside from Pu are the major interfering species and compromise its direct measurement with all the abovementioned measurement techniques. Matrix components can cause further interferences and thereby often prevent a direct preparation of the sample for subsequent measurements themselves.^2^ As a result, Pu isotopes must be chemically separated from other actinide elements and matrix components prior to their analysis. The separation procedures are typically carried out using extraction chromatography^9–13^ and ion exchange chromatography^14,15^ after dissolution of samples by acid digestion^16^ or alkali fusion.^17–20^ The analytical procedures often comprise several chromatographic columns together with precipitation steps, which makes them tedious and time-consuming. Due to the very rich redox chemistry of early actinides, many of these chromatographic approaches rely directly on an oxidation-state adjustment to induce selectivity during the separation procedures.^9,11,12,14,15^ This is usually done using classical reducing and oxidizing agents. Electrolysis offers an interesting alternative to these more traditional approaches since it provides direct control of the oxidation states. Flow-through electrolysis with large specific surface area electrodes is of special interest since it enables fast electrolysis and convenient coupling with standard chromatographic methods.^21–24^ However, in such settings, the instability of the electrogenerated oxidation state can be problematic if it is further reduced/oxidized back to its original oxidation state. This behavior has been observed while coupling flow-through electrolytic cells with ion exchange or extraction chromatography, for example, the separation of Ce and Eu from other lanthanides.^25–28^ Therefore, introducing oxidation-state-selective chemical functionalities directly on the electrode provides a straightforward way of reliably controlling the oxidation state of the analyte right next to these selective chemical features.

In the past, direct modification of packed carbon fiber electrodes by impregnation with extractants such as trioctylphosphine oxide (TOPO) or di­(2-ethylhexyl)­phosphoric acid (HDEHP) has been used for the separation of early actinides^29–31^ in different oxidation states and for the separation of Bk.^32^ Surface modification of carbon fiber electrodes with a Nafion cation-exchange polymer also enabled concomitant oxidation state control and sorption for the separation of carrier-free amounts of Ce, as well as the late actinide elements No and Md.^33–35^ However, these two approaches still suffer from the same drawbacks as ion exchange and extraction chromatography as they rely on expensive extractants or ion exchange polymers, which may leach out during the separation and thus complicate the further processing of the sample (e.g., the preparation of high-quality α-sources for α-spectrometry^15,36^ or filaments for TIMS^17,37^ ). In addition, chemical modification of the electrodes must be done prior to placing them in the electrolytic flow cell. Meanwhile, a direct electrochemical functionalization of the electrode surface within the flow cell with covalently bound chemical features would circumvent all of these problems. The modification of a glassy carbon (GC) electrode surface by anodization was shown to induce the accumulation of U, Pu, or Th and could be used for their preconcentration and separation from interfering elements and matrix components prior to ICP-MS analysis.^38–42^ Although this approach might be used for the analysis of long-lived radioisotopes of these elements, it does not, however, provide a quantitative accumulation of the analyte and it is not ideal for the pre-analytical separation of shorter-lived isotopes (e.g., ^232^U, ^238,241^Pu), which are measured by radiometric methods such as α-spectrometry or LSC. In that case, high recoveries of the analyte are needed to provide acceptable counting times.

Therefore, we propose here the use of anodized carbon fibers as a working electrode for the quantitative separation of Pu isotopes from other actinide elements, as well as matrix impurities. The high surface area of this cost-effective material is well-suited for the quantitative accumulation in a flow-through electrolysis mode and allows for high recoveries with ultratrace amounts of actinide elements. The flow electrolysis approach proposed here has substantial advantages with respect to extraction or ion exchange chromatographic methods since it prevents the introduction of chemical impurities from the resins and oxidizing/reducing agents. With this, one omits the addition of contaminants and facilitates the further processing of a sample. It also provides a fast, single-step separation, while several columns are sometimes needed to fully remove interfering radionuclides.^2,16,18–20,37^ As is the case for its classically used chromatographic counterparts, the proposed approach enables the preconcentration of the analyte, allows for very efficient matrix removal, and is readily automated. Flow electrolysis can also be easily combined in series with chromatography, making it a perfect complementary tool for pre-analytical separation procedures. Consequently, the aim of this study was to implement a flow-through electrolysis approach for the separation and analysis of early actinides. In this work, a procedure for the anodization of carbon fiber felt was developed, and the resulting anodized carbon fiber working electrodes were characterized in detail with X-ray photoelectron spectroscopy (XPS) and infrared spectroscopy (IR). The selectivity of the thus prepared working electrodes for different oxidation states of the actinides was evaluated by using Pu as a model analyte. Unraveling the underlying mechanism responsible for the accumulation of Pu on the electrode surface under applied potential allowed for further development and optimization of the separation procedure toward a reliable analysis of Pu isotopes by α-spectrometry. The developed analytical procedure was applied for the measurement of ^238^Pu as well as ^239,240^Pu in digested solid samples (i.e., wipe tests, ground ceramic, or sludge) and evaluated by comparison with well-established chromatographic separation methods.

## Experimental Section

### Chemicals and Materials

All reagents were of analytical grade, obtained from Merck, VWR, or Thermo Fisher Scientific, and were used as received. All solutions were prepared by using 18.2 MΩ·cm^–1^ deionized water (Milli-Q®, Merck Millipore). Tracer solutions were prepared by diluting stock solutions with certified radioactivity concentrations of ^238,239,240,242^Pu (Eckert & Ziegler; AEA Technology), ^241,243^Am (Eckert & Ziegler), and ^244^Cm as well as ^236^U (AEA Technology). The anion exchange resin AG®1-X2 was obtained from Bio-Rad, and the TEVA extraction resin was obtained from Triskem International. The carbon fiber felt electrodes were purchased from EC Frontiers.

### Flow Electrolysis Setup

The VF2 flow-through cell used for flow electrolysis was obtained from EC Frontiers. A schematic of this flow-through electrolysis cell is shown in Figure 1. The flow cell working electrode (WE) consisted of an 18 mm diameter carbon fiber felt disk of 5 mm thickness with a surface area of ≈ 1900 cm^2^. The Pt counter electrode (CE) and the reference electrode (RE) were located in a separate compartment to avoid any contamination from counter electrode reactions. This compartment was filled with 1 M KNO_3_. An Ag/AgCl/3 M KCl electrode (0.209 V *vs* standard hydrogen electrode) was used as the RE, and all potentials reported herein are given in reference to this electrode. The flow cell was operated with a Gamry interface 1010E potentiostat, which allowed for carrying out flow electrolysis at a controlled potential. The solution was circulated through the flow cell with a peristaltic pump (IPC-16, Ismatec). In addition, the experimental setup enabled the insertion of an extraction chromatography cartridge after the flow cell exit in order to further capture selected outgoing ions.

**Figure 1:**
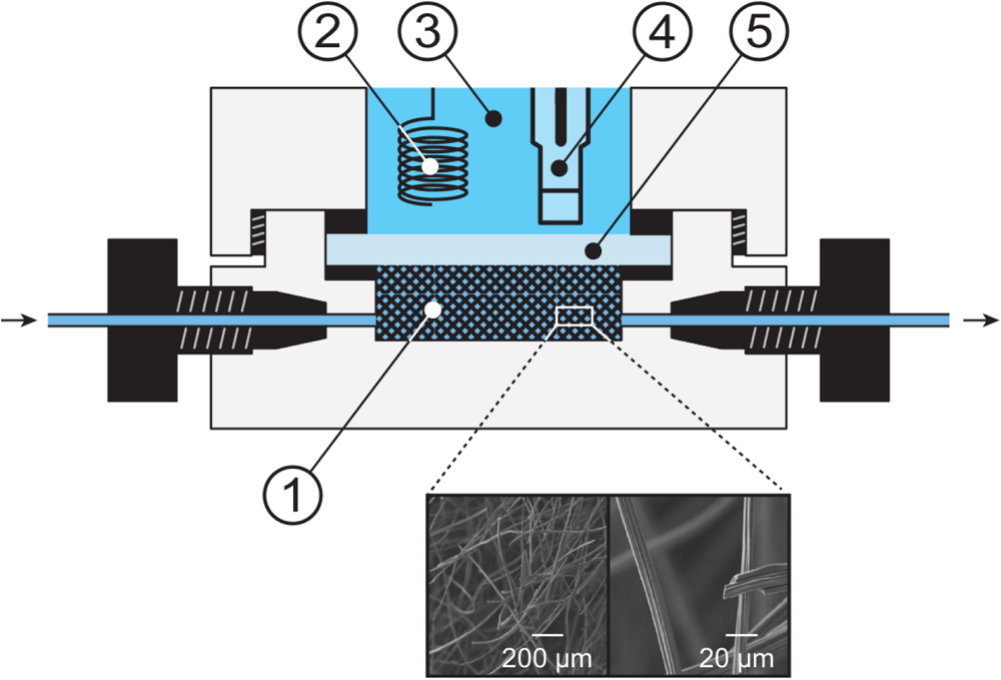
Cross-sectional scheme of the flow-through electrolysis cell with (1) the carbon fiber felt working electrode, (2) the Pt counter electrode, (3) the electrolyte compartment for the counter and reference electrodes, (4) the Ag/AgCl/3 M KCl reference electrode, and (5) the porous glass separator; scanning electron microscope images of the carbon felt at different magnifications are shown below.

### Anodization of the Working Electrode

The carbon fiber felt provided with the flow cell (EC Frontiers) was cleaned by ultrasonication in ethanol for 10 min, followed by a thorough rinse with 18.2 MΩ·cm^–1^ deionized water, before being soaked in 1 M KNO_3_ and placed in the flow cell. It was then anodized by cycling its potential between 1.5 V and 2.5 V at 50 mV·s^–1^ for 900 cycles in 1 M HNO_3_ at a 1 mL·min^–1^ flow rate. The obtained anodized carbon fiber felt could either be used directly as the WE for flow electrolysis experiments or removed from the flow cell, rinsed with deionized water, and dried for 2 h in an oven at 80 ^◦^C for further characterization.

### Characterization of the Working Electrode

The characterization by XPS was carried out with a monochromatic XR 50M Al Kα1 radiation source (1486.7 eV) operated at 12.5 kV and 24.1 mA. The emitted photoelectrons were measured with a PHOIBOS 150 electron analyzer. The working pressure inside the XPS vacuum chamber was kept below 2 × 10^–9^ mbar during spectra acquisition. Analysis and fitting of the XPS spectra were performed using the CasaXPS software (Casa Software Ltd), and the parameters proposed by Biesinger were used for the peak fitting of the C1s high-resolution spectra.^43^ All IR spectroscopy measurements were conducted in diffuse reflectance mode (DRIFTS) by using a Vertex 70 FT-IR spectrometer from Bruker, equipped with a Praying Mantis mirror unit (Harrick) and a DTGS detector. Individual IR spectra were obtained by averaging 5000 interferograms at a spectral resolution of 4 cm^−1^ and a scanner velocity of 10 kHz. The sample was loaded into a DRIFTS sample cup and inserted into the mirror unit, which was purged with dry N_2_.

### Sample Pretreatment

The method was tested for the determination of radioactivity concentrations of Pu isotopes in three different types of solid samples, namely (1) a wipe test from the primary circuit of a nuclear power plant, (2) ground ceramic tiles from the wall of a radioactive wastewater tank, and (3) sludge from a contaminated retention basin.

The wipe test was digested in acids according to a procedure described in detail elsewhere.^24^ In short, the wipe test was placed in a mixture of 10 mL of conc. HNO_3_, 2 mL of conc. HCl, and 1 mL of conc. H_2_SO_4_, heated to 200–250 °C for 2 h, and subsequently to ≈ 400 ^◦^C on a heating plate for several hours until total dissolution (> 4 h). The solution was then evaporated to dryness at 500 °C and taken up in 8 M HNO_3_. Aliquots from this solution were taken for further analysis, spiked with 0.2 Bq of the ^242^ Pu yield tracer, evaporated once more to dryness, and taken up in the appropriate molarity of HNO_3_ for further separation.

The ceramic and sludge samples were chemically digested using a lithium borate alkali fusion procedure. Prior to the fusion, each sample was dry ashed in a muffle furnace, initially for 1 h at 100 °C, followed by a slow ramping of the temperature until 800 °C where the sample was kept for 6 h. After cooling to room temperature, a portion of each sample (0.1–0.5 g) was weighed in a Pt/Ag crucible, spiked with 0.2 Bq of ^242^Pu yield tracer, and mixed with the Spectroflux B flux reagent (LiBO_2_:Li_2_B_4_O_7_, 80:20 w/w-%). The subsequently carried out automated fusion procedure has been described elsewhere.^19,20^ Briefly, the crucible containing the sample was heated up to 1065 °C in a Claisse LeNeo (Malvern Panalytical) apparatus, and the produced molten salt was poured into 4.5 M HNO_3_ and further dissolved by subsequent heating to 200 °C. The SiO_2_ particles were removed by flocculation with polyethylene glycol 6000 (PEG) and subsequent filtration. Aliquots of the obtained solution could then be evaporated to dryness and taken up in the appropriate molarity of HNO_3_ for further separation.

### Chemical Separation of Pu

For the flow electrolysis separations, the treated samples (see procedure outlined in the Sample Pretreatment section above) were dissolved in conc. HNO_3_, evaporated to dryness, and further dissolved in 10 mL of 1 M HNO_3_. The solution thus obtained was injected into the flow cell at 0.46 mL·min^–1^ while applying 1.2 V to the previously anodized WE in order to accumulate Pu. The WE was then rinsed with 10 mL of 1 M HNO_3_ at 0.46 mL·min^–1^ to remove other actinides as well as matrix components. The separated Pu fraction was finally recovered in 1 M HNO_3_ at a 2 mL·min^‑1^ flow rate by successively applying −0.2 V for 10 min before cycling the potential from −0.5 V to 0 V at 1 V·s^–1^ for 900 scans.

The Pu separation was also carried out using a well-established ion exchange chromatography procedure based on the use of the strong base anion exchange resin AG®1-X2.^14,15^ 2 g of resin were slurry packed in a chromatographic column with an inner diameter of 10 mm and conditioned with 8 M HNO_3_. The sample was dissolved in 8 M HNO_3_, and the careful addition of H_2_O_2_ was carried out under gentle heating (≈ 100 °C) in order to ensure the stabilization of Pu in the oxidation state +4.^2,14^ The obtained solution was injected into the column, which led to the elution of U and transplutonium isotopes. Th isotopes were washed out using 20 mL of 10 M HCl, while Pu was eluted and recovered in a final step by reduction with 20 mL of 0.1 M HI and 9 M HCl solution.

### Analysis by α-Spectrometry

All α-sources were prepared by electrodeposition on stainless steel planchets from a phosphate buffer according to the method of Bajo and Eikenberg.^36^ The eluates obtained after chromatography were mineralized in conc. HNO_3_ and H_2_O_2_ in order to remove organic impurities from the resin prior to electrodeposition. This particular step was not necessary for the fractions collected during separation by flow electrolysis. Measurements were performed using an Alpha Analyst^TM^ α-spectrometer (Mirion Technologies) equipped with PIPS detectors (Passivated Implanted Planar Silicon), with detection efficiencies between 34% and 41%. The sources prepared from model mixtures of different radiotracers were measured for 24 h, while those obtained from the wipe test, as well as the ceramic and sludge samples, were measured for 100 h. The analysis of the α-spectra was carried out using the Genie^TM^ 2000 software package (Mirion Technologies).

## Results and Discussion

### Electrode Anodization

Anodization of carbon-based electrode materials in H_2_SO_4_ or HNO_3_ is a well-known method to introduce oxygen-containing chemical functionalities on the predominantly sp^2^-hybridized carbon surface.^44,45^ The affinity of anodized GC for U and Pu has been demonstrated in the past.^38,40^ The reproducibility of the Pu separation obtained with the carbon fiber felt WE used in the present study proved to be highly dependent on the anodization treatment. The procedure proposed for the anodization of GC^38,40^ failed to yield reproducible results when applied to carbon fibers. After optimization, it was found that longer and harsher anodization conditions are required to attain reproducible results with carbon fiber WEs. This was achieved by cycling the carbon fiber felt potential between 1.5 V and 2.5 V in 1 M HNO_3_ (see Experimental Section). The anodized carbon fiber felt obtained was characterized to understand the structural changes resulting from the anodization treatment. No morphological changes were observed during the investigation with a scanning electron microscope. However, surface characterization by means of XPS and DRIFTS revealed clear changes with the introduction of oxygen-containing functional groups. The corresponding XPS survey spectra (see Figure 2a) clearly show the appearance of the O1s level peak upon anodization of the carbon fiber felt. This indicates the formation of oxygen-containing functionalities on the surface. The absence of a signal of N1s at ≈ 400 eV indicates that no NO_3_^–^ is retained on the anodized surface and that no nitrogen-containing functional groups nor intercalation compounds are formed during the treatment of the WE.

**Figure 2:**
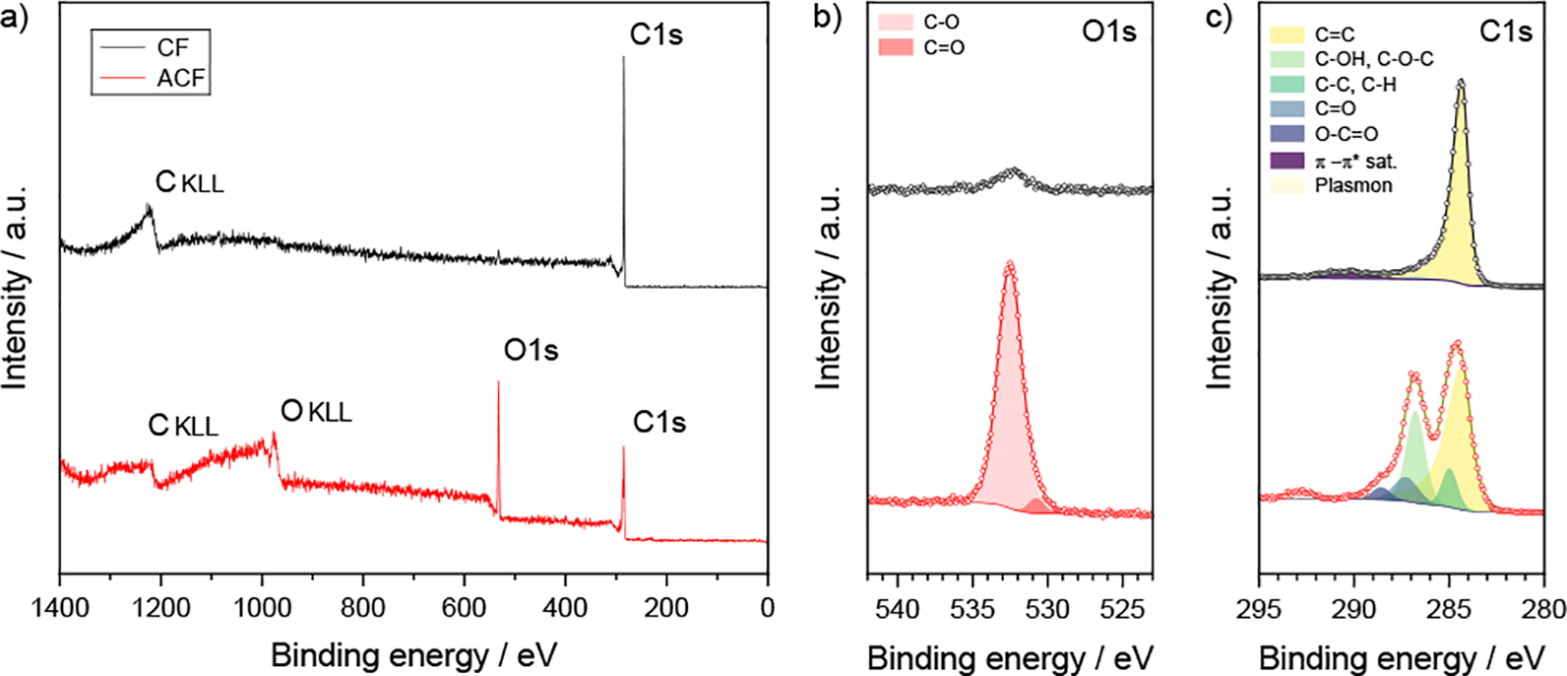
Investigation of the elemental composition on the surface of the carbon fiber felt electrode (CF, black spectra, top) and the anodized carbon fiber felt (ACF, red spectra, bottom) by means of XPS; the XPS survey spectra are presented in (a), and the high-resolution spectra of the O1s and C1s regions are presented in (b) and (c), respectively. The measured high-resolution spectra (circles) are shown with the corresponding fits (solid lines) and individual components from spectrum deconvolution (shaded peaks).

The observed XPS spectra agree well with previously reported results and further support the fact that potentials of ≥ 2 V are indeed necessary to introduce oxygen-containing moieties on carbon fiber surfaces in HNO_3_ media.^46^ Spectrum deconvolution of the high-resolution C1s peak reveals that the carbon fiber surface is mainly graphitic before anodization (see Figure 2c), as shown by the dominating asymmetric C=C peak at 284.4 eV and its π-π^∗^ shake-up satellite at 290.5 eV.^43,47,48^ The appearance of C–C and C–H groups (285.0 eV) as well as the vanishing of the π-π^∗^ satellite after anodization (see Figure 2c) suggests the partial loss of the sp^2^ graphitic structure upon anodic treatment. In addition, the C1s signal shows a chemical shift due to the formation of carbon-oxygen bonds (see Figure 2c). This results in the appearance of three components, which were attributed to C–OH and C–O–C groups (286.8 eV; main contribution), C=O groups (287.3 eV), and O—C=O groups (288.6 eV).^43,47,48^ The presence of additional features at higher binding energies in the case of the anodized carbon fibers may be attributed to plasmon losses. Analysis of the high-resolution O1s peak observed on anodized carbon fibers (see Figure 2b) shows a main feature that can be associated with oxygen singly bound to aliphatic carbon (532.5 eV) with a minor contribution attributed to oxygen doubly bound to carbon in aromatic structures (531.0 eV).^47,49^ A weak signal is also measured at the O1s peak for the carbon fiber before anodization; this is most likely a result of the oxygen-containing groups typically present on the edges of graphene planes in graphitic structures.^44,45^ XPS spectra were measured at different locations in the carbon fiber felt, revealing consistent results, thereby confirming the homogeneous functionalization of the surface throughout the electrode.

The DRIFTS spectrum of the anodized fiber felt exhibits a broad band extending above 2000 cm^–1^ region with a maximum at 3494 cm^–1^ and two bands at 1718 cm^–1^ and 1573 cm^‑^^1^, all of which are absent in the spectrum of the bare carbon fiber felt (see Figure 3). The broader band can be attributed to the presence of hydrogen-bonded –OH-groups, while the band at 1718 cm^–1^ can be assigned to C=O entities from carbonyl or carboxyl functional groups.^47,50,51^ Meanwhile, the signal at 1573 cm^–1^ may originate from aromatic C=C stretches from unoxidized regions.^47,50,51^ These results are in good agreement with the XPS investigation and confirm that the anodization procedure indeed introduces hard oxygen donor functional groups on the surface of the carbon fiber felt. The oxygen moieties introduced seem to be mainly hydroxyls and ethers but also partially carbonyls, carboxyls, or esters (see Figure 4).

**Figure 3:**
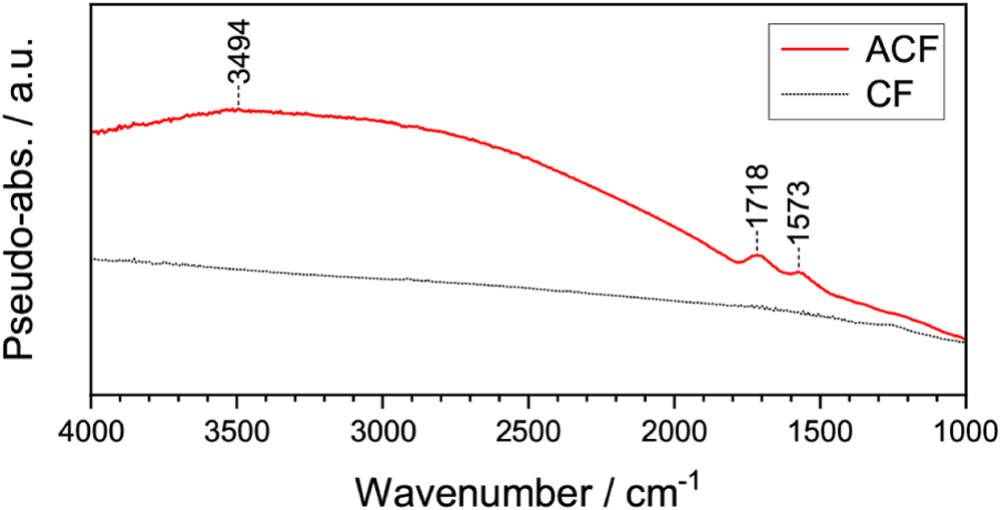
Analysis of the functional group of carbon fiber felt (CF) and anodized carbon fiber felt (ACF) with DRIFTS.

**Figure 4:**
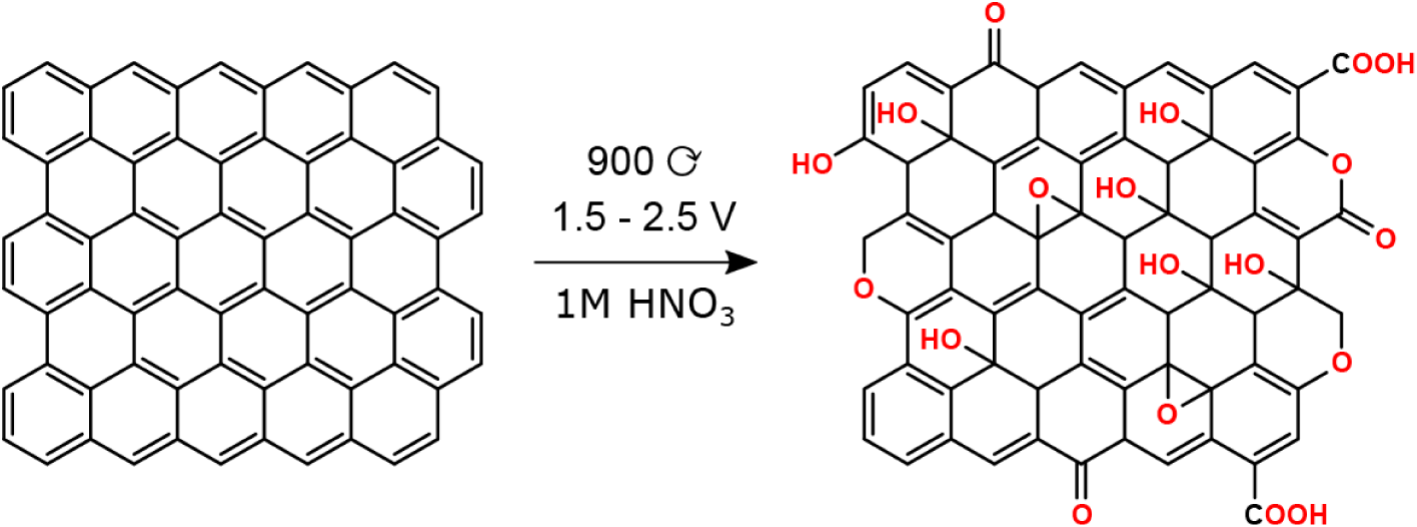
Possible functional groups introduced on the surface of the carbon fiber felt by the anodization procedure employed.

### Flow Electrolysis

After anodization of the carbon fiber felt, trace amounts of Pu can be retained in the flow cell at high applied potential (e.g., 1.2 V) and released again at low potential (e.g., −0.2 V). Under these conditions, other actinides such as U, Am, or Cm are not retained in the flow cell, thus affording their direct separation from Pu. In acidic solutions, Pu is mainly found in the Pu­(III) and Pu­(IV) oxidation states but may also be present as Pu­(V) or Pu­(VI).^52^ The electrochemical reaction involving the Pu­(III)/Pu­(IV) couple has been extensively studied at carbon fiber electrodes in various media,^22^ and the formal potential observed herein is in good agreement with the values obtained for retention of Pu on anodized GC.^40^ Therefore, the main electrochemical reaction triggered at the WE is attributed to the 1-electron reduction/oxidation of the Pu­(III)/Pu­(IV) couple. In order to be retained on the electrode surface, Pu needs to be present as Pu­(IV), which can be stabilized at 1.2 V from the Pu­(III)/Pu­(IV) mixtures. Pu­(IV) is a harder Lewis acid than Pu­(III), Pu­(V), as well as Pu­(VI) (i.e., present as *trans*-dioxo PuO_2_^+^ and PuO_2_^2+^ plutonyl ions in aqueous solutions, respectively) and tends to have stronger interactions with hard Lewis base ligands in solution in the following order: Pu­(IV) > Pu­(III) ≈ Pu­(VI) > Pu­(V).^52^ This trend is generally preserved for all actinide elements (An) and typically translates to stronger adsorption of the An­(IV) oxidation state on surfaces containing hard Lewis base moieties. For instance, stronger sorption of harder lanthanide/actinide ions such as Th­(IV) or Pu­(IV) compared to softer ones such as U­(VI), Np­(V), Am­(III), or Eu­(III) is observed on graphene oxide materials, which is structurally closely comparable to the surface of the anodized carbon fibers used in this study.^53–56^ On graphene oxide, the sorption of hard Th­(IV) was shown to be caused by the formation of strong inner-sphere surface complexes with oxygen donor atoms from carboxylic acid and hydroxyl surface functionalities.^57^ Therefore, a similar behavior may explain the stronger interactions of Pu­(IV) with the hard oxygen-containing moieties present on the anodized carbon fiber surface and its resulting retention. This also explains why Pu is not retained on a non-anodized carbon fiber felt in the flow cell, even upon application of 1.2 V to the WE. For the procedure to be effective, the starting oxidation state of Pu must be +3 or +4. Therefore, the samples were heated and evaporated to dryness in conc. HNO_3_ in order to ensure the reduction of any Pu­(V)/Pu­(VI) to Pu­(III)/Pu­(IV)^58^ before re-dissolution in the appropriate electrolyte. Here, the reduction was made possible by the species formed during the thermal decomposition of HNO_3_, especially NO_2_.^59,60^ The addition of H_2_O_2_ ^2,14^ or calcination in H_2_SO_4_ ^15,61^ may provide alternative pathways for the quantitative reduction to Pu­(III)/Pu­(IV) prior to flow electrolysis. A large anodic overpotential of 1.2 V subsequently afforded quantitative electrolysis of Pu­(III) and ensured full retention of Pu as Pu­(IV). Therefore, no further efforts were made to optimize the applied potential. The developed procedure could then be used for the quantitative retention of Pu­(IV) and its subsequent release by reduction to Pu­(III) at −0.2 V, with an additional cycling of the potential between −0.5 V and 0 V. A typical elution profile obtained during the separation of ^242^Pu from ^244^Cm in a model mixture of tracers is shown as an example in Figure 5.

**Figure 5:**
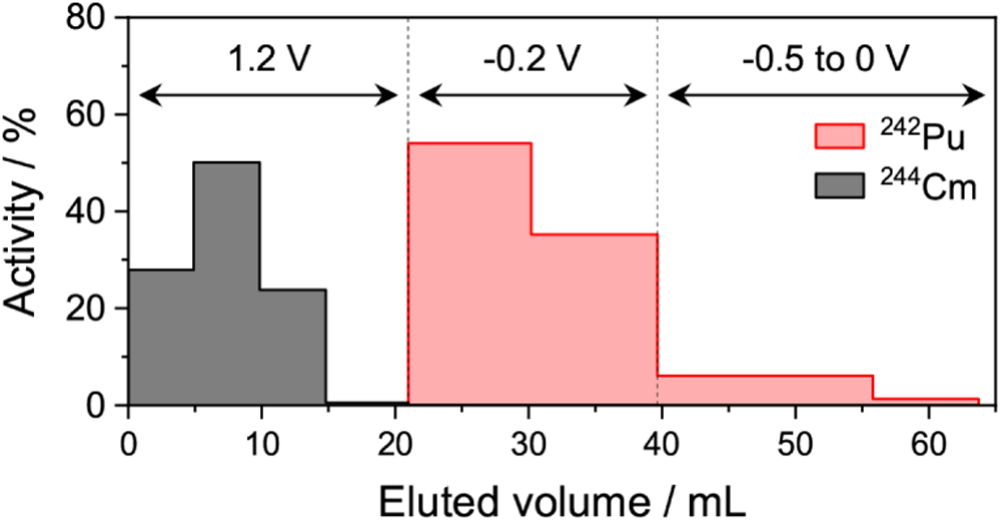
Elution profile obtained during separation of 0.5 Bq ^242^Pu (red) from 0.5 Bq ^244^Cm (black) by flow electrolysis in 1 M HNO_3_. The radioactivity concentrations of ^242^Pu and ^244^Cm were measured by α-spectrometry. The fractions collected at 1.2 V were obtained using a 0.26 mL·min^–1^ flow rate; the others were collected at 2 mL·min^–1^.

Electrochemical oxidation of Pu­(IV) to higher oxidation states +5 and +6 proceeds only at high overpotentials and is highly irreversible due to the associated formation of multiple Pu−O bonds. Therefore, when starting from a solution containing only Pu­(III) and Pu­(IV), no electrochemical oxidation beyond Pu­(IV) is expected at 1.2 V. For instance, no further oxidation was observed at this potential in HClO_4_ media.^23,62^ However, several studies have recently reported the electrochemical oxidation of Pu­(IV) to Pu­(VI) in HNO_3_ media with a Pt WE.^63–65^ Therefore, further experiments were conducted to ensure that only Pu­(IV) was indeed adsorbed on the anodized WE and that no higher oxidation states were formed. A Pu tracer solution was flushed through the flow cell, and Pu was accumulated at 1.2 V in 0.5 M HNO_3_. In the following, Pu was released from the flow cell by flushing the WE with 3 M HNO_3_ (i.e., leading to the elution of Pu without changing the potential on the WE; see paragraph below) to a TEVA chromatography cartridge coupled in series with the flow cell. TEVA extraction resin has a strong affinity for Pu­(IV) under these conditions but does not retain other oxidation states of Pu.^12^ About 94% of the Pu flushed through the TEVA resin was indeed retained in the cartridge, thereby confirming that Pu­(IV) is the main form accumulated on the anodized carbon fiber felt at 1.2 V.

The influence of the electrolyte type and concentration, as well as the flow rate, on the Pu recovery yield was also investigated in order to further optimize the separation conditions. Recovery yields of nearly 100% could be obtained in 0.5 M and 1 M solutions of both HNO_3_ and HCl (see Figure 6a and 6b). In both cases, the yields progressively decreased with higher acid concentrations but showed overall better recoveries for HCl. Additional experiments in H_2_SO_4_ media show that it was not possible to accumulate any Pu (see Figure 6c). The differences observed in the recovery yields as functions of the electrolyte types and their concentrations show a decrease in the following sequence: HCl > HNO_3_ > H_2_SO_4_. This trend reflects the increase in the complexation strength of the acid anions toward Pu­(IV) from Cl^–^ to SO_4_^2–^.^52^ In addition, the recovery yields are different at similar pH values with different electrolytes. This supports the idea that the difference of drop in recovery yields at high molarities is mainly driven by the competing pathway of anion complex formation. The competition for the binding sites with H^+^ may also partly contribute to the decrease in recovery yields at high acid concentrations. As a result, Pu previously accumulated in 0.5 M HNO_3_ at 1.2 V could be stripped simply by rinsing the flow cell with, for example, 3 M HNO_3_, while the potential was kept fixed at the same time at 1.2 V.

**Figure 6:**
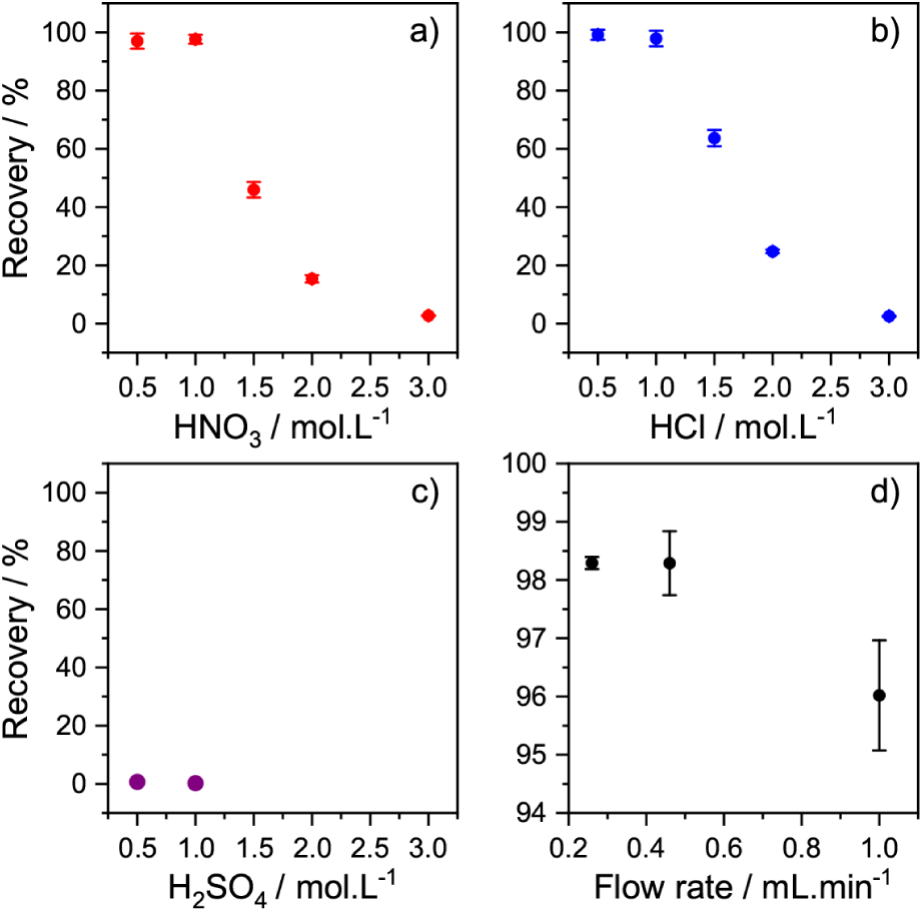
Recovery yield of 0.5 Bq ^242^Pu obtained by separation using flow electrolysis as functions of (a) the concentration of HNO_3_, (b) the concentration of HCl, (c) the concentration of H_2_SO_4_, and (d) the flow rate used (as obtained in 1 M HNO_3_); all data points of (a), (b), and (d) are expressed as the average and standard deviation for *n* = 3 experiments, whereas for (c), only one experiment was performed. The recovery yields were obtained by α-spectrometry. Error bars may be smaller than the symbol.

The recovery yields were slightly higher at flow rates below 0.5 mL·min^–1^, although generally good performance is also achieved at 1 mL·min^–1^ (see Figure 6d). With these well-optimized parameters, fast separations of Pu from other actinides are readily achieved, allowing for interference-free measurements of Pu isotopes by α-spectrometry. Typical α-spectra before and after flow electrolytic separation of various Pu isotopes from ^236^U, ^241,243^Am, and ^244^Cm tracers in a model mixture are displayed in Figure 7. The procedure used here was a compromise between procedural speed and separation efficiency. However, even longer rinsing times and lower flow rates could be used if better decontamination of Pu from U, Am, and Cm isotopes is in fact necessary.

**Figure 7:**
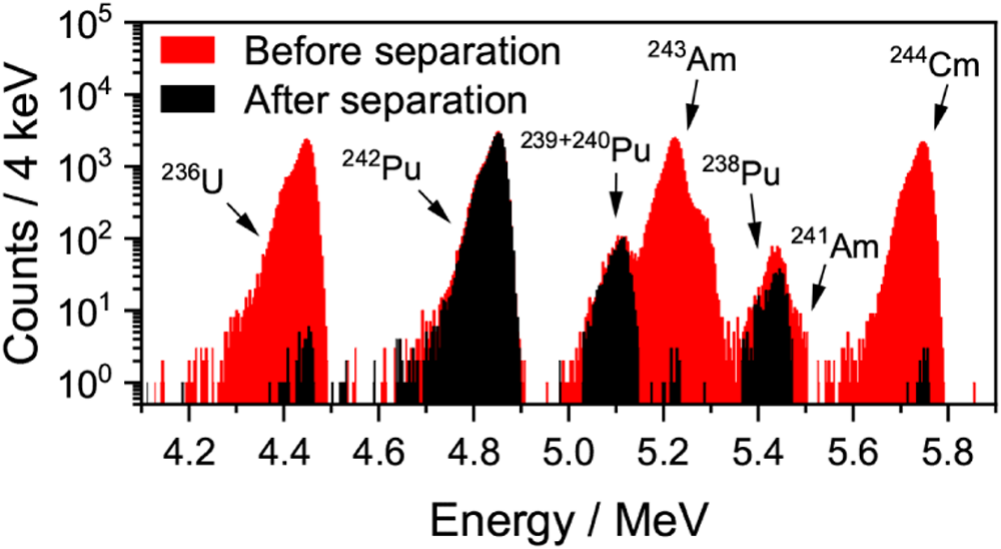
α-spectra of a mixture of ^238,239,240,242^Pu, ^236^U, ^241,243^Am, and ^244^Cm before (red) and after (black) separation of Pu by flow electrolysis in 0.5 M HCl at 0.46 mL·min^–1^ with an anodized carbon fiber electrode; both spectra were obtained after 48 h live time counting. A quantity of ≈ 0.5 Bq of ^242^Pu, ^236^U, ^243^Am, and ^244^Cm was used during this experiment, with traces of ^238,239,240^Pu and ^241^Am.

Although the main focus of this work was directed toward Pu separations, a similar procedure was also tested for U in 1 M HNO_3_. In the case of U accumulation and release, the potentials used were −0.15 V and 1.2 V, respectively. These settings were based on the results obtained by Pretty and coworkers with anodized GC.^38^ At −0.15 V U is reduced from U­(VI) (as UO_2_^2+^) to U­(IV),^22^ which is a hard Lewis acid and is therefore retained on the anodized carbon fiber felt. This further supports the proposed mechanism regarding the retention of Pu­(IV) on the anodized carbon fiber surface.

### Application to Radioactive Samples

The suitability of the flow-through electrolysis method for complex samples was further investigated in the case of three different samples. A wipe test from the primary circuit of a nuclear power plant as well as ceramic shreds from a radioactive wastewater tank and sludge from a contaminated retention basin were analyzed for their Pu isotopes. After the appropriate destruction of the solid sample by acid digestion or by lithium borate fusion (see Sample Pretreatment), the Pu isotopes had to be separated from the bulk of the matrix in order to prepare appropriately thin sources for α-spectrometry. In addition, the separation was mandatory to remove any other α-emitters, such as other actinide isotopes present in the sample, which may interfere during the measurement. Evaporation to dryness and further evaporation after the addition of conc. HNO_3_ were found to be necessary in order to achieve good results with flow electrolysis. This was attributed to the reduction of any high oxidation state of Pu to Pu­(III) or Pu­(IV) (see Flow Electrolysis). All flow electrolytic separations were carried out in 1 M HNO_3_ due to the satisfactory results obtained during previous experiments with model mixtures of various tracers and the absence of any precipitation from these samples. Chromatographic separations with anion exchange or extraction resins were used as a benchmark for direct comparison with the flow electrolysis approach evaluated here. All findings regarding the ^238^Pu and ^239+240^Pu radioactivity concentrations, as well as the recovery yields of the ^242^Pu tracer, are reported in Table 1.

**Table 1:** Radioactivity Concentrations of ^238^Pu and ^239+240^Pu as Well as the Recovery Yields, as Determined with the ^242^Pu Yield Tracer, in Various Samples Using Flow Electrolysis (FE) and Chromatography (C) for *n* = 3 with the Standard Deviation as the Uncertainty

Matrix	Separation method	^238^Pu (Bq·g^–1^)	239 + 240Pu (Bq·g^–1^)	^242^Pu recovery (%)
Wipe test	FE	174.5 ± 2.1	118.9 ± 1.5	64.4 ± 10.5
	C	177.4 ± 1.7	120.2 ± 1.7	65.1 ± 15.9
Ceramic	FE	11.7 ± 0.5	245.3 ± 1.4	61.6 ± 2.5
	C	11.9 ± 0.6	249.1 ± 1.9	80.7 ± 26.3
Sludge	FE	7.2 ± 0.1	28.2 ± 0.5	27.1 ± 4.5
	C	6.8 ± 0.1	27.9 ± 0.1	91.9 ± 1.0

All radioactivity concentrations of ^238^Pu and ^239 + 240^Pu of the three samples, as determined after separation by flow electrolysis and chromatography, agree well within the given uncertainties. Comparably larger differences of < 3% between the two methods are obtained for ^239 + 240^Pu in the ceramic sample and for ^238^Pu in the sludge sample. The recovery yields seem to be dependent on the matrix, with similar values for the wipe test sample but a notable difference for the sludge sample, where the achieved recoveries are markedly lower for flow electrolysis than for chromatography. Even though high recoveries are desired to get more reliable measurements, this did not impede the proper quantification of the analyte. The lower recoveries obtained with the sludge samples suggest that some matrix components interfere more strongly with the Pu­(IV) adsorption in the case of more complex samples featuring a broader variety of ions. This is most likely a result of competition for the electrode binding sites (fouling) with ions originally present within the sample matrix. Additionally, the ions originating from the lithium borate fusion procedure are likewise competing for these binding sites and, thus, contribute to a general decrease in recoveries. This was confirmed by tracer experiments, which reveal lower recovery yields in the presence of lithium borate. Therefore, acid digestion should be considered an alternative to circumvent this problem and potentially improve the recovery yields. Last, the PEG employed during sample preparation was also found to significantly impair recovery yields when conducting similar tracer tests in its presence. In fact, PEG is known to covalently bond to carbon electrode materials at the anodic potential used here for Pu­(IV) accumulation.^66^ A resulting PEG layer may impede the electron transfer needed for the electrochemical oxidation/reduction of Pu and its accumulation/release. Therefore, the SiO_2_ removal step with PEG should be avoided during sample preparation whenever possible. As a consequence, an additional cleaning step was found to be necessary to remove residual actinides or matrix components out of the flow cell between each separation with the ceramic or sludge samples. This was done by flushing the setup with 3 M HNO_3_ at 2 mL·min^–1^ with potential cycling between −0.5 and 0 V. Despite the lower recoveries when going toward more complicated sample matrices, the developed method provides a fast, single-step separation method for Pu analysis, which leads to a processing time comparable to that of a single-column chromatography separation. Additional advantages of flow electrolysis are (1) a seamless removal of the sample matrix and (2) the avoidance of organic impurities introduced into the investigated sample as a consequence of leaching of the extraction or ion exchange resins. Hence, there is no need for mineralization after the recovery of Pu, whereas this operation must be routinely carried out following a separation by chromatography to meet the requirements for the preparation of α-sources by electrodeposition.^15,36^ Therefore, the presented approach also allows for the preparation of high-quality α-sources, which feature excellent spectroscopic resolutions during α-spectrometric measurements (e.g., ≈ 25 keV full width at half-maximum for the main α-line of ^242^Pu at *E*_α_ = 4.902 MeV). Last, flow electrolysis can be easily coupled with extraction chromatography to directly collect other non-retained trivalent transuranium elements of interest, such as Am or Cm, from the effluent using, e.g., a DGA extraction cartridge.^13^

## Conclusion

The *in situ* anodization of a carbon fiber felt electrode allowed a high surface area electrode to be homogeneously functionalized with different oxygen donor groups. The thus prepared anodized carbon fibers enable the concomitant control of the Pu oxidation state by flow electrolysis and the selective binding of Pu­(IV), as well as its subsequent release through electroreduction to Pu­(III). This effect could be successfully leveraged for the single-step separation of Pu isotopes from other actinide elements, including U, Am, and Cm, as well as matrix components. A fast analytical procedure was developed for the separation and analysis of Pu isotopes by α-spectrometry and compared to a traditional chromatographic separation approach. The procedure allowed accurate determination of Pu isotopes in arguably difficult sample matrices, such as ceramics or sludge. This showcases the matrix tolerance of the presented separation approach by flow electrolysis and its applicability for the analysis of samples in the context of the control of nuclear facilities, their decommissioning, and environmental monitoring. In addition, the method is expected to be easily adapted, for, e.g., ^241^Pu measurements by LSC or ^239^Pu and ^240^Pu measurements with ICP-MS (and ^239^Pu/^240^Pu ratios with, for example, TIMS). The postulated retention mechanism suggests that other actinides in their +4 oxidation states should be likewise retained on the anodized surface, as observed during additional tests carried out with U. Therefore, a similar method holds promise for the pre-analytical separation of U isotopes, for which the different chemical conditions of accumulation and release have yet to be studied in more detail in the future. The flow electrolytic separation approach may as well be adopted to other actinide or lanthanide elements known to exist in the +4 oxidation state (e.g., Ce, Np, or Bk). Further characterization of the electrode surface may also lead to a better understanding of the retention mechanism of An­(IV) on the anodized carbon fiber electrode and ultimately to improved separation conditions.
